# Allergic bronchopulmonary candidiasis: A review of the literature and a case report

**DOI:** 10.1186/s12890-022-01921-3

**Published:** 2022-04-07

**Authors:** Martin Scurek, Eva Pokojova, Martina Doubkova, Kristian Brat

**Affiliations:** 1 Department of Respiratory Diseases, University Hospital Brno, Jihlavska Str. 20, 62500 Brno, Czech Republic; 2 Faculty of Medicine, Masaryk University, Brno, Czech Republic; 3 International Clinical Research Center, St. Anne’s University Hospital, Brno, Czech Republic

**Keywords:** Allergic bronchopulmonary candidiasis, Allergic bronchopulmonary mycosis, *Candida albicans*, Pulmonary infiltrates

## Abstract

**Background:**

Allergic bronchopulmonary candidiasis (ABPC) is an uncommon clinical syndrome associated with immune hypersensitivity to *Candida* species.

**Case presentation:**

The case presentation describes a 58-year-old man with acute respiratory failure and bilateral lung infiltrates. Due to high inflammatory markers and a chest X-ray indicating lung infiltration, he was initially treated for pneumonia with combined antibiotics. Despite comprehensive treatment at the ICU, the patient’s clinical status deteriorated rapidly, and further investigations provided a rare diagnosis of ABPC. After several days of combined corticosteroid and antifungal therapy, we observed rapid clinical improvement and subsequent resolution of the pulmonary infiltrates.

**Conclusion:**

This case report presented a rare case of ABPC mimicking bilateral pneumonia and acute respiratory failure. Our case highlighted the importance of prompt corticosteroid and antifungal treatment initiation as it resulted in rapid clinical improvement and a near complete reversal of the bilateral lung infiltrates.

## Background

Allergic bronchopulmonary candidiasis (ABPC) is a lower respiratory tract disease caused by a hypersensitivity-mediated reaction to the *Candida* species and is part of a broader clinical entity named the allergic bronchopulmonary mycosis (ABPM). ABPM is commonly associated with bronchial asthma and cystic fibrosis, and it is caused by a wide range of environmental fungi, the most common being *Aspergillus fumigatus*, *Candida albicans*, and *Bipolaris* species [1]. Several criteria must be fulfilled for a diagnosis of ABPM, and these criteria were recently revised by the International Society of Human and Animal Mycology (ISHAM) [2]. Briefly, two major and two minor criteria must be met, including (major criteria) elevated serum level of total IgE and cutaneous hypersensitivity to fungal antigen or elevated specific IgE against the fungal pathogen and (minor criteria) serum IgG antibodies against the fungal antigen, a radiographic finding or elevated blood eosinophil count (Table 1) [2]. The treatment of ABPM is based on oral administration of prednisone for several weeks or months while the role of antifungal treatment remains controversial [1]. While allergic bronchopulmonary aspergillosis (ABPA) is most frequent among the group of ABPMs, ABPC is a rarely reported condition.

**Table 1:** Diagnostic criteria for ABPA proposed by the International Society of Human and Animal Mycology (ISHAM) [2]

Predisposing conditions:
Bronchial asthma, cystic fibrosis
Obligatory criteria (both should be present):
Type I *Aspergillus* skin test positivity (immediate cutaneous hypersensitivity to *Aspergillus* antigen) or elevated IgE levels against *A. fumigatus*
Elevated total IgE levels (˃ 1000 UI/ml)^a^
Other criteria (at least two of three):
Presence of precipitating or IgG antibodies against *A.fumigatus* in serum
Radiographic pulmonary opacities consistent with ABPA^b^
Total eosinophil count ˃ 500 cells/µl in steroid naïve patients (may be historical)

^a^If the patient meets all other criteria, an IgE value < 1000 UI/ml may be acceptable

^b^The chest radiographic features consistent with ABPA may be transient (i.e., consolidation, nodules, tram-track opacities, toothpaste/finger-in-glove opacities, fleeting opacities) or permanent (i.e., parallel line or ring shadows, bronchiectasis and pulmonary fibrosis)

Herein, we report on a severe manifestation of ABPC featuring respiratory failure requiring ICU admission and prolonged hospitalization.

## Case presentation

**Fig. 1:**
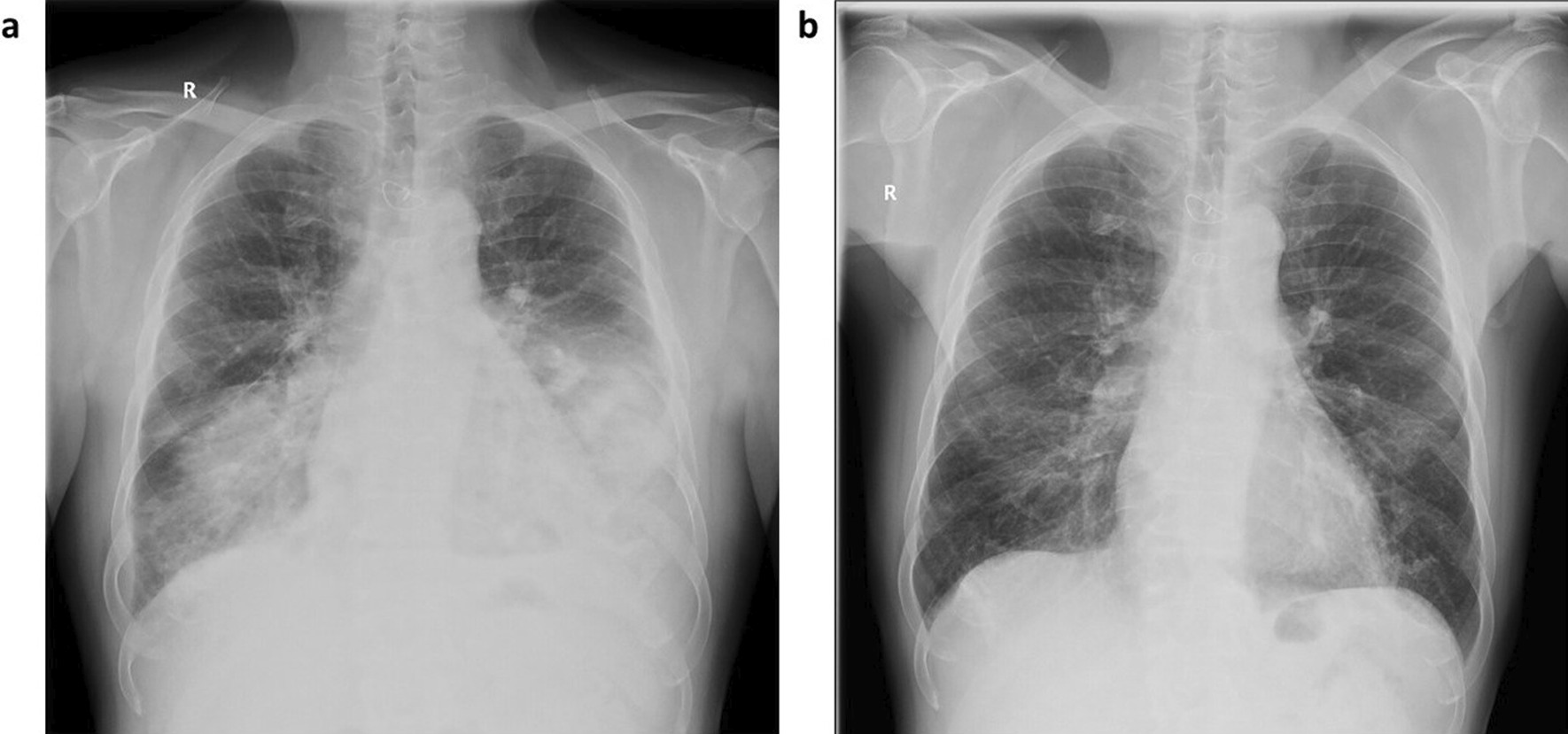
Chest X-ray. **a** at hospital admission showing extensive bilateral infiltrates predominantly in basal segments of the lungs; **b** two weeks after corticosteroid administration with almost complete resolution of bilateral lung infiltrates

In November 2018, a 58-year-old man was admitted to the ICU with acute respiratory failure. The patient presented with dyspnea, cough, chest pain and acute respiratory insufficiency. Arterial blood gases revealed isolated hypoxemia (p_A_O_2_: 46 mmHg) without hypercapnia or acidosis. He had a history of multiple diseases, including asthma-COPD overlap, diabetes mellitus, ischemic heart disease, arterial hypertension and atrial fibrillation. The patient had been a non-smoker for seven years but had a smoking history of 15 pack-years. Bilateral crackles were present, predominantly in the basal segments of the lungs during chest auscultation; there were no other relevant findings upon physical examination. Blood tests showed an elevated white blood cell count with high blood inflammatory markers (C-reactive protein was 178 mg/L) and the absolute eosinophil count was in the normal range (410 eosinophils/µL). Finally, a chest x-ray revealed infiltration of both lower lobes of the lungs (Fig. 1a).

The findings suggested pneumonia and we treated the patient with a combination of antibiotics (clarithromycin and amoxicillin-clavulanate) and supplemental oxygen therapy. After one week, the antibiotic treatment had no effect and the patient developed a high fever. A chest radiograph indicated pulmonary infiltrate progression, although we had switched to piperacillin-tazobactam. Sputum cultivation was repeatedly positive for *C. albicans*. The material (sputum) was inoculated with a calibrated 10 µL loop (manufacturer: Copan, Italy) on Sabouraud’s agar with antibiotics (manufacturer: Conda, Spain). The plates were incubated for 5 days at temperatures ranging between 35 and 37 °C. The obtained cultures were inoculated on chromagar (manufacturer: Biolife, Italy) and subsequently identified by the MALDI-TOF method (manufacturer: Bruker, Germany). Thus, we initiated fluconazole treatment at a higher dosage (200 mg twice daily for 28 days). We didn´t perform pulmonary function tests concerning patient dyspnea and increasing oxygen dependency.

**Fig. 2:**
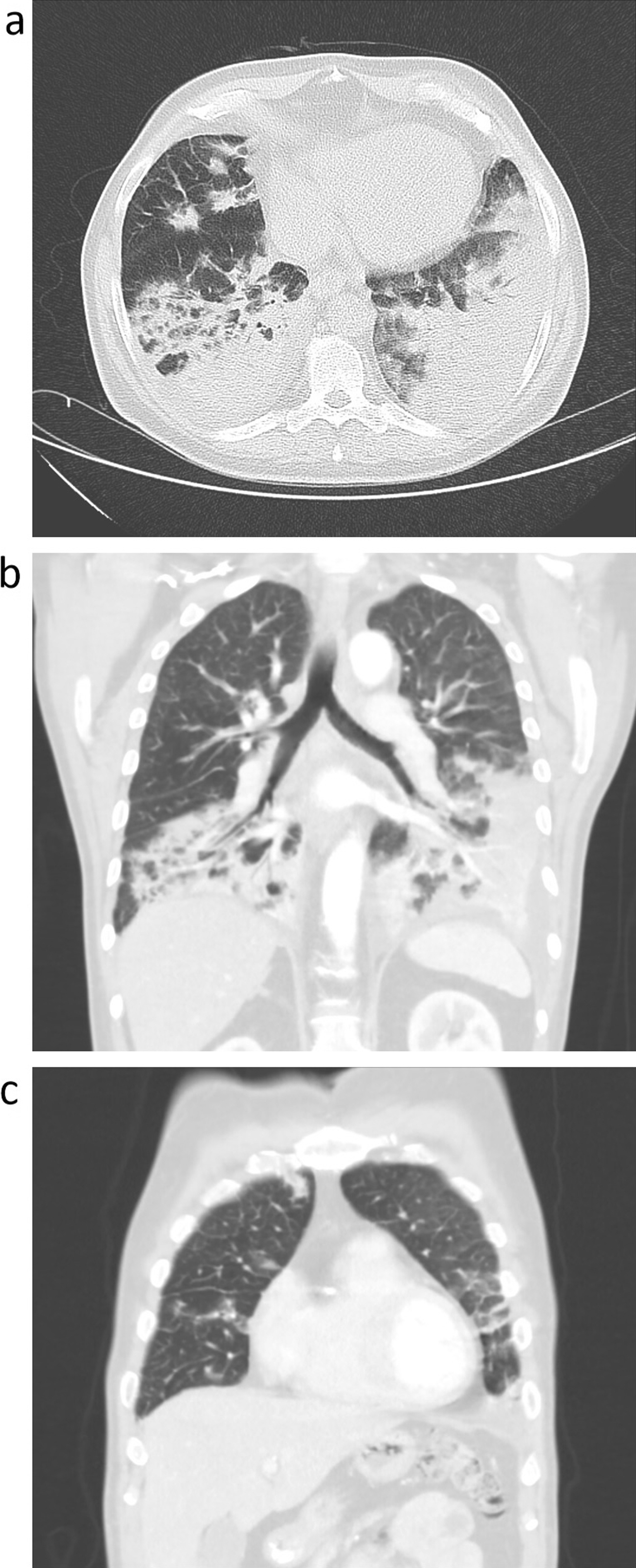
High-resolution computed tomography images: **a** computed tomography axial scan showing almost complete consolidation of both lower lobes; **b** computed tomography coronal scan showing almost complete consolidation of both lower lobes; **c** computed tomography coronal scan showing several smaller consolidations in the right upper and middle lobes and in the left upper lobe

His general condition remained poor, as he had high fever, fatigue, and progressive respiratory failure into hypercapnia (pH: 7.46; pCO_2_: 51 mmHg; pO_2_: 44 mmHg; HCO_3_: 35.7 mmol/L; Sat. O_2_: 81%). Therefore, we performed a CT scan and bronchoscopy. The CT scan showed almost complete consolidation of both lower lobes (Fig. 2a, b) and several smaller consolidations in the rest of the lungs (Fig. 2c). There were no pathological macroscopic findings during the bronchoscopy. The cultivation of bronchoalveolar lavage fluid (BALF) was negative for the presence of any bacteria. We obtained the levels of galactomannan (GM) and (1,3)-β-d-glucan (BD) from both BALF and blood serum, and all results were within normal ranges; exactly BALF GM was 0.12 (normal range < 0.5), BALF BD 0.0 pg/mL (normal range < 0.08 pg/mL), blood GM 0.07 (normal range < 0.5) and blood BD 0 pg/mL (normal range < 0.08 pg/mL). However, significant increase in eosinophils was present in the BALF differential count (6% of eosinophils; normal range ˂2%) and high total IgE serum levels (IgE 1575 IU/mL; normal range < 150 IU/mL) were found. Thus, we tested for *Aspergillus* and *Candida*-specific IgE. The results were negative for the specific IgE for *Aspergillus*; however, they were strongly positive for *Candida*-specific IgE (3.17 IU/mL; normal range ˂ 0.35 IU/mL). The institutional laboratory of the University Hospital Brno used the Immulite® 2000/2000XPi immunoassay system (manufacturer: Siemens Healthineers AG, Erlangen, DE), the kits catalog numbers were M3L4 and M5L4. A skin prick test for *Candida* (and other fungi) was not undertaken due to its non-availability at our center.

Having confirmed our suspicion regarding ABPC, we immediately started treatment with prednisone (0.5 mg/kg/day). We also continued fluconazole therapy to reduce the fungal antigen burden. After two weeks of corticosteroids, we observed rapid regression of the pulmonary infiltrates (Fig. 1b). Consequently, both clinical status and respiratory failure started to improve rapidly; thus, we discontinued oxygen therapy. The peripheral blood eosinophil count decreased from 410 to 0 cells/µL within 4 days since the corticosteroid treatment was initiated and remained in normal range (varying between 0 and 80/µL cells) both during the course of corticosteroid therapy and during the following 3 years. After more than five weeks of hospital stay, the patient was discharged with no need for ventilatory support and no exertional dyspnea. Altogether, we administered fluconazole for four weeks and corticosteroids for eight weeks. After the initial course of prednisone (6 weeks at 0.5 mg/kg/day), we tapered the dose for two weeks before discontinuing treatment.

In summary, a definitive diagnosis of ABPC was established based on the clinical presentation, peripheral blood and BALF eosinophilia and elevated serum total IgE and specific anti-*C. albicans* IgE. Moreover, rapid clinical improvement occurred in response to the combined corticosteroid and antifungal therapy.

## Discussion and conclusion

Various fungal infections may occur in the lungs, each of them may predispose to allergic sensitization and the fungal allergens should be examined in patients with pulmonary infiltrates [3–5].

ABPM is a clinical syndrome associated with immune responses to several fungi. The most common form of ABPM and the first to be described, allergic bronchopulmonary aspergillosis (ABPA), was initially noted in patients with asthma, allergic sensitization to *A. fumigatus* and lung infiltrates with a potential to progress to fibrotic lung disease [6]. The second most common ABPM is ABPC, which was described for the first time in the early 1980s by several authors, who listed *C. albicans* as the causative agent [7, 8]. Several ABPC cases with serious clinical manifestations were reported during the last few years [9, 10].

While ABPA is widely known due to its association with cystic fibrosis and asthma, ABPMs caused by other fungi are understudied and possibly underdiagnosed worldwide due to inadequate clinical awareness. In reports on the world incidence of ABPMs other than ABPA, India accounted for 47% of globally reported cases, most of which were attributed to *C. albicans*, followed by Japan, with nearly 16% of all cases [1].

There is no definite biomarker of ABPM; thus, diagnosis is indirect and relies on the fulfillment of several diagnostic criteria, all of which are not specific to allergic mycotic diseases.

The most recent classification was proposed by the ISHAM group in 2013. According to this classification, cystic fibrosis and asthma are predisposing factors. There are two major diagnostic criteria (obligatory)—cutaneous hypersensitivity to *Aspergillus* antigen (or elevated specific IgE against *A. fumigatus*) and elevated serum level of total IgE. Finally, two of the three additional criteria (serum IgG antibodies against *A. fumigatus*, radiographic findings and total eosinophil count of more than 500 cells/µL) must be met (Table 1) [2]. This classification was initially proposed for ABPA; however, ABPM can be diagnosed in a similar manner by inserting sensitization to another fungal agent [1].

Definite diagnosis of ABPC can be hard to achieve due to unspecific biomarkers. The first of ISHAM’s obligatory requirements concerns total IgE, which can be elevated across a wide range of allergic diseases; furthermore, there is no definitive consensus on the cut-off value. ISHAM guidelines use the cut-off value of IgE > 1000 IU/mL, which is higher than those used by other authors; however, this high value helps distinguish ABPA from asthma with fungal sensitization [2]. A recent Indian study proposed an even higher cut-off value (2347 IU/mL), to better distinguish ABPM further [11]. Total IgE should be monitored as a follow-up during treatment, and in well-treated patients, we should see a drop in its value over time.

The second mandatory requirement is specific IgE against *C. *albicans or other fungi as a sign of a hypersensitive reaction to one of these antigens. Nevertheless, due to the common presence of fungi in our environment, it can also reflect mere environmental exposure or colonization [12]. The same problem crops up with the presence of *Candida* in sputum cultures. Its presence is mandatory for documenting a patient’s hypersensitivity reaction; however, many asthma patients have positive *Candida* cultures due to inhaled corticosteroid overuse (colonization), not because they present with ABPC.

Blood eosinophil count is a marker of eosinophilic inflammation in an organism, and it is usually done for all types of allergic diseases. However, one-quarter of ABPA patients have normal eosinophil counts; in addition, there is a poor correlation between eosinophil count and disease activity [13]. The eosinophil count is unreliable due to the interference of corticosteroids. Systemic corticosteroids, as well as high doses of inhaled corticosteroids, reduce blood eosinophils [14, 15].

Assessment of serum and bronchoalveolar lavage fluid GM and BD has become a diagnostic standard for invasive fungal disease, mainly in immunocompromised patients [16, 17]. There is still surprisingly little data on the diagnostic yield of GM and BD in ABPM. However, from available data, it seems that serum GM is usually within normal ranges in patients with ABPA [18]. Similarly, in our case, the blood and BALF levels of GM and BD were all within normal ranges. These findings impose that the examination of serum and BALF levels of GM and BD may be of certain importance as increased levels may confirm invasive fungal infection while within-normal-range levels may suggest the presence of ABPM/ABPA/ABPC.

When ABPM is considered, a critical role is played by radiographic methods. The most common radiographic finding for acute-stage ABPM is transient pulmonary infiltrates, known as “fleeting opacities,” but it is important to note that the chest x-ray may be normal in up to 50% of cases [19]. On CT scans, ABPM can present a wide range of findings, but the most typical ABPM presentation features central bronchiectasis and high-attenuating mucus in the airways [20]. At the same time, the presence of bronchiectasis indicates a predisposition to recurrent relapses [20]. It is also possible to observe consolidations, segmentary collapses, cavities, bullae, pleural effusions and other anomalies [21].

The main goals of ABPC treatment are immunosuppression and eradication of the causative agent. The eradication of the causative antigen is difficult due to its ubiquitous nature. The most critical treatment involves systemic corticosteroids. Across the literature, there is no consensus on the dosing scheme, but a high dosage of prednisone (0.5 mg/kg/day) should be administered for at least two weeks and then tapered for several weeks or even months [22]. This treatment sequel should result in prompt resolution of pulmonary infiltrates and to a decline in total serum IgE levels. Similarly, opinions vary on antifungal therapy and there is no definitive agreement regarding the most effective molecule or dosage, yet alone the duration of treatment [1]. Furthermore, there is a lack of robust evidence-based data on the efficacy of antifungal therapy in ABPM, in general, and there are genuine concerns about raising azole resistance during its long-term administration [23]. A few authors have also reported on inhalation of antifungal drugs [24]. Consistent data regarding the use of inhaled corticosteroids during ABPM treatment are lacking; however, from the available data, even high doses of inhaled corticosteroids were not beneficial compared to standard of care and corticosteroids should not be used as first-line therapy [25]. Furthermore, even with precise therapy, the risk for exacerbation after therapy withdrawal is indisputable [22, 23].

An emerging novel therapeutic option for ABPM is the treatment with omalizumab. Li et al. reported reduction of symptoms, decreased exacerbation rate and reduction of corticosteroid doses, as well as decreased serum IgE [26]. The omalizumab treatment had good safety profile however there was no improvement in lung function [26].

We reported a rare case of ABPC presenting as extensive bilateral lung infiltrates and respiratory failure. In our patient´s case, ABPC mimicked bilateral pneumonia. As there was no clinical improvement after empirical antibiotics, additional tests for alternative diagnoses were performed, establishing the diagnosis of ABPC. The corticosteroid and antifungal treatment resulted in rapid improvement of the patient´s condition within a few weeks.

In conclusion, our case underlines that ABPC can be a life-threatening disease that can present with bilateral lung infiltrates and severe respiratory insufficiency. Precise diagnosis and early corticosteroid and antifungal therapy initiation appears to be the treatment of choice, as prompt clinical improvement is expected.

## Data Availability

All data generated or analyzed during this study are included in the manuscript.

## Abbreviations

ABPA: Allergic bronchopulmonary aspergillosis
ABPC: Allergic bronchopulmonary candidiasis
ABPM: Allergic bronchopulmonary mycosis
BALF: Bronchoalveolar lavage fluid
BD: (1,3)-β-d-glucan
CT: Computed tomography
GM: Galactomannan
ICU: Intensive care unit
IgE: Immunoglobulin E
ISHAM: International Society of Human and Animal Mycology
MALDI-TOF: Matrix assisted laser desorption ionization—time of flight
